# New ways for an old cation

**DOI:** 10.1007/s00424-020-02394-1

**Published:** 2020-05-25

**Authors:** Martin Diener

**Affiliations:** Institute for Veterinary Physiology and Biochemistry, Justus Liebig University Giessen, Frankfurter Str. 100, 35392 Giessen, Germany

In this issue of *Pflügers Archiv European Journal of Physiology*, Liebe et al. describe the properties of bTRPV3 (bovine transient receptor potential vanilloid type 3) channels expressed in model systems such as HEK-293 cells or *Xenopus* oocytes. By using a combination of different methods such as pH- and voltage-sensitive microelectrodes or patch-clamp recordings, they demonstrate that this channel is highly permeable for NH_4_^+^. This cation is produced in large amounts in the forestomach during the degradation of proteins and non-protein nitrogen compounds by microbes living in symbiosis with the ruminant [8]. With a pKa value of 9.2, NH_4_^+^ is the overwhelming form in the chemical equilibrium between NH_3_ (ammonium) and NH_4_^+^ (ammonia) at the slightly acidic pH values within the ruminal content. The expression of this channel in the bovine ruminal epithelium makes it a good candidate as the (or one of the) transport proteins for NH_4_^+^. Ammonia is rapidly absorbed from the forestomach, converted to urea in the liver and recycled into the forestomach as substrate for microbial protein synthesis or excreted via the urine. Probably, TRPV3 is also the molecular basis for the long-known divalent-sensitive cation conductance found in the forestomach epithelium [10].

Transport of NH_4_^+^/NH_3_ has up to now thought to be mediated by simple non-ionic diffusion of the gas NH_3_ or by renal rhesus-associated glycoproteins, members of the SLC42 solute transporter family able to transport both NH_4_^+^ and NH_3_ [1]. Furthermore, many K^+^ channels and transporters accept NH_4_^+^ instead of K^+^ due to the similar hydrated radius (1.45 Å) of both ions [6]. This property has been elegantly used in transport physiological studies to measure e.g. activity of Na^+^-K^+^-2 Cl^−^-transporters (NKCC) via bumetanide-sensitive acidification in the presence of extracellular NH_4_^+^ [5, 7].

The present study of Liebe et al. now adds a new player, bTRPV3, to the list of transporters involved in ammonia transport. When regarding the biophysical properties of this unselective cation channel, bTRPV3 shows a remarkably high permeability for N-methyl-D-glucamine^+^ (NMDG^+^), which is often used as ‘impermeant’ ion during cation substitution experiments. Despite the large molecular size of NMDG^+^, which is estimated to cover a volume of 6 Å × 6 Å × 12.5 Å [4], fitting of shifts in zero-current potential during ion replacement experiments to the Goldman-Huxley-Katz equation revealed a permeability for NMDG^+^ of about 45 % in comparison with that for Na^+^. Due to these properties and its permeability even for divalent cations such as Ca^2+^, demonstrated by the same research group [9], it is well possible that beside ammonia absorption, also other absorptive functions of the ruminal epithelium may involve TRPV3.

The study of Liebe et al. has relevance for several fields. Production of climate gases such as N_2_O from cattle urine (starting from urea produced during the hepatic metabolism of NH_4_^+^ absorbed by the forestomach) is a severe environmental problem [2]. So, basic knowledge about the physiological mechanisms of ruminal NH_4_^+^ absorption delivering the substrate for urea production is urgently needed. A further outlook of the present study is the question whether TRPV3 or similar channels may be involved in the absorption of NH_4_^+^ from the large intestine such as caecum or colon, where also significant amounts of ammonium are absorbed. Healthy individuals protect their central nervous system from NH_3_/NH_4_^+^ by hepatic conversion into urea; any hepatic failure can therefore lead to encephalopathy. As many members of the superfamily of TRP channels can be pharmacologically activated or inhibited by different drugs or natural compounds (see [3]), these channels might be promising candidates for new therapeutic strategies.
