# Correction to: New ways for an old cation

**DOI:** 10.1007/s00424-021-02593-4

**Published:** 2021-07-01

**Authors:** Martin Diener

**Affiliations:** Institute for Veterinary Physiology and Biochemistry, Justus Liebig University Giessen, Frankfurter Str. 100, 35392 Giessen, Germany

**Correction to: Pflügers Archiv—European Journal of Physiology (2020) 472:669–670**

**https://doi.org/10.1007/s00424-020-02394-1**

The article “New ways for an old cation”, written by Martin Diener, was originally published Online First without Open Access. After publication in volume 472, issue 6, page 669–670 the author decided to opt for Open Choice and to make the article an Open Access publication. Therefore, the copyright of the article has been changed to © The Author(s) 2020 and the article is forthwith distributed under the terms of the Creative Commons Attribution 4.0 International License, which permits use, sharing, adaptation, distribution and reproduction in any medium or format, as long as you give appropriate credit to the original author(s) and the source, provide a link to the Creative Commons licence, and indicate if changes were made. The images or other third party material in this article are included in the article’s Creative Commons licence, unless indicated otherwise in a credit line to the material. If material is not included in the article’s Creative Commons licence and your intended use is not permitted by statutory regulation or exceeds the permitted use, you will need to obtain permission directly from the copyright holder. To view a copy of this licence, visit http://creativecommons.org/licenses/by/4.0.

The original article has been corrected.

**Open Access** This article is licensed under a Creative Commons Attribution 4.0 International License, which permits use, sharing, adaptation, distribution and reproduction in any medium or format, as long as you give appropriate credit to the original author(s) and the source, provide a link to the Creative Commons licence, and indicate if changes were made. The images or other third party material in this article are included in the article's Creative Commons licence, unless indicated otherwise in a credit line to the material. If material is not included in the article's Creative Commons licence and your intended use is not permitted by statutory regulation or exceeds the permitted use, you will need to obtain permission directly from the copyright holder. To view a copy of this licence, visit http://creativecommons.org/licenses/by/4.0/.

